# Rust Infection of Black Poplar Trees Reduces Photosynthesis but Does Not Affect Isoprene Biosynthesis or Emission

**DOI:** 10.3389/fpls.2018.01733

**Published:** 2018-11-27

**Authors:** Franziska Eberl, Erica Perreca, Heiko Vogel, Louwrance P. Wright, Almuth Hammerbacher, Daniel Veit, Jonathan Gershenzon, Sybille B. Unsicker

**Affiliations:** ^1^Department of Biochemistry, Max Planck Institute for Chemical Ecology, Jena, Germany; ^2^Department of Entomology, Max Planck Institute for Chemical Ecology, Jena, Germany; ^3^Zeiselhof Research Farm, Pretoria, South Africa; ^4^Department of Zoology and Entomology, Forestry and Agricultural Biotechnology Institute, University of Pretoria, Pretoria, South Africa; ^5^Technical Service, Max Planck Institute for Chemical Ecology, Jena, Germany

**Keywords:** biotrophic pathogens, disease, isoprenoids, MEP pathway, non-mevalonate pathway, plant hormones, Salicaceae, stomatal conductance

## Abstract

Poplar (*Populus* spp.) trees are widely distributed and play an important role in ecological communities and in forestry. Moreover, by releasing high amounts of isoprene, these trees impact global atmospheric chemistry. One of the most devastating diseases for poplar is leaf rust, caused by fungi of the genus *Melampsora*. Despite the wide distribution of these biotrophic pathogens, very little is known about their effects on isoprene biosynthesis and emission. We therefore infected black poplar (*P. nigra*) trees with the rust fungus *M. larici-populina* and monitored isoprene emission and other physiological parameters over the course of infection to determine the underlying mechanisms. We found an immediate and persistent decrease in photosynthesis during infection, presumably caused by decreased stomatal conductance mediated by increased ABA levels. At the same time, isoprene emission remained stable during the time course of infection, consistent with the stability of its biosynthesis. There was no detectable change in the levels of intermediates or gene transcripts of the methylerythritol 4-phosphate (MEP) pathway in infected compared to control leaves. Rust infection thus does not affect isoprene emission, but may still influence the atmosphere *via* decreased fixation of CO_2_.

## Introduction

Poplar (*Populus* spp.) trees are deciduous, woody plants that are widely distributed in the northern hemisphere (Stanton et al., 2010; Isebrands and Richardson, 2014). In their natural habitat, which consists of floodplain forests and riverbanks, they are of significant ecological importance as host plants for an enormous diversity of microbes, insects, and other organisms. Apart from that, poplar trees have gained increased economic attention in recent years as fast growing sources of wood, plywood, paper, and biofuels when grown in short-rotation coppices (Karp and Shield, 2008). The fast growth, which also favors agronomical use, combined with the availability of the sequenced genome (Tuskan et al., 2006) and the possibility of clonal reproduction, makes this tree an excellent model organism for woody plant research. However, natural populations as well as plantations of poplar regularly suffer from severe infections by biotrophic rust fungi (Polle et al., 2013). *Melampsora* rusts are among the most devastating diseases for poplars worldwide (Pei and Shang, 2005; Wan et al., 2013; Štochlová et al., 2016). An epidemic outbreak of rust disease can cause drastic losses over 50% in biomass production^1^ (Gérard et al., 2006), premature defoliation and even mortality in young stands (Aylott et al., 2008; Benetka et al., 2011; Polle et al., 2013). Upon rust infection, poplar trees activate the salicylic acid (SA) defense signaling pathway (Azaiez et al., 2009; Eberl et al., 2018), as in many other plants infected by biotrophic pathogens (Glazebrook, 2005) leading to an enhanced production of phenolic secondary metabolites and the expression of pathogenesis-related proteins (Miranda et al., 2007; Rinaldi et al., 2007; Azaiez et al., 2009; Chen et al., 2014; Ullah et al., 2017). However, studies connecting rust infection with primary physiological processes are rare, even though it can be assumed that the biotrophic lifestyle of the rust fungus will unbalance the host’s primary metabolism dramatically (Berger et al., 2007; Voegele and Mendgen, 2011). Rust infection also modifies volatile emissions of poplar, leading to increased release of monoterpenes (C_10_) and sesquiterpenes (C_15_) (Eberl et al., 2018). In addition, poplar trees emit large amounts of isoprene (C_5_). This small volatile hydrocarbon is emitted by a number of plant species, most of them woody. Poplar trees are amongst the strongest isoprene emitters known (Logan et al., 2000; Laothawornkitkul et al., 2009). In the atmosphere, isoprene is involved in the formation of ozone and hydroxyl radicals, and hence plays an important role in atmospheric chemistry (Sharkey et al., 2008). Even though isoprene is emitted in considerable amounts that exceed the emission of all other biogenic volatiles (Guenther et al., 1995), its biological function is still not well understood. Isoprene is hypothesized to increase the thermotolerance of plants, protect against ozone and oxygen radicals, and act as a “safety valve” for dissipating energy under high light conditions (Loreto and Velikova, 2001; Sharkey et al., 2008; Laothawornkitkul et al., 2009).

The biosynthesis of isoprene occurs in the chloroplasts *via* the methylerythritol 4-phosphate (MEP) pathway (Logan et al., 2000), an alternate route for isoprenoid production to the mevalonate (MVA) pathway, which is located in the cytoplasm. The MEP pathway is present in higher plants, algae and some bacteria, but not in fungi and animals (Hemmerlin et al., 2012). Both pathways produce dimethylallyl diphosphate (DMADP) and isopentenyl diphosphate (IDP), the universal building blocks for isoprenoid formation (Rodríguez-Concepción and Boronat, 2015). The regulation of both pathways occurs at different levels, ranging from transcriptional control of genes encoding biosynthetic enzymes to post-translational modifications of pathway enzymes (Hemmerlin, 2013). The MEP pathway is tightly connected to photosynthesis, not only spatially by sharing the same compartment, but also metabolically. The MEP pathway consumes metabolic intermediates, energy and reducing equivalents drawn directly from the light and dark reactions; in return it produces the photosynthetic pigments, chlorophylls and carotenoids, and phytohormones (Hemmerlin et al., 2012). One of these hormones is abscisic acid (ABA) which controls stomatal opening and mediates responses to drought stress (Acharya and Assmann, 2009). However, ABA is also known to be involved in defense reactions against biotic stressors, but usually acts as an antagonist to SA-mediated signaling cascades (Ton et al., 2009; Cao et al., 2011).

In the past, most studies on isoprene emission have focused on its biosynthesis, its effects on atmospheric chemistry and its involvement in plant interactions with the abiotic environment. For poplar, 40% of all the articles on isoprene emission since 1990 studied its relation to abiotic environmental factors^2^ (January 2018), while only 3% studied its relation to biotic environmental factors (Brilli et al., 2009; Müller et al., 2015; Jiang et al., 2016).

We therefore investigated the effect of the pathogenic rust fungus *Melampsora larici-populina* on isoprene emission from black poplar trees (*Populus nigra*). We monitored temporal changes of photosynthesis and isoprene emission during infection and investigated underlying physiological mechanisms by analyzing aspects of leaf chemistry, physiology, and transcriptional changes.

## Materials and Methods

### Experimental Material

Black poplar (*P. nigra* L.) trees were grown from hardwood cuttings (photosynthesis experiment and isoprene experiment) or softwood cuttings (transcriptome experiment) obtained from different tree genotypes growing in a common garden in Isserstedt, Germany (50°57′28.5″N 11°31′17.4″E). One genotype was used for the transcriptome experiment as well as for the photosynthesis and isoprene measurements, whereas two different genotypes were additionally used for photosynthesis measurements. The cuttings were potted in 2 l- pots, grown in the greenhouse (18/20°C, night/day, relative humidity 60%, natural light with 9–14 h photoperiod, supplement light for 12 h, SON-T Agro; Philips, Andover, MA, United States) and transferred to an environmental chamber [18/20°C, night/day; relative humidity 60%; photoperiod 16 h, MT 400 (Eye, Uxbridge, United Kingdom)] 2 days before the onset of the experiment. All three experiments (photosynthesis experiment, isoprene experiment and transcriptome experiment) were conducted separately at different time points but under the same temperature and humidity conditions. All trees were used ca. 4 months after potting and had reached a height of about 0.5 m. Trees used for photosynthesis and isoprene measurement did not show any noticeable shoot growth at time of experiment.

Uredospores of the biotrophic poplar leaf rust fungus (*M. larici-populina* Kleb.) were obtained from naturally infected black poplar trees growing in the above-mentioned common garden. The identity of the fungus was verified by using specific primers for the internal transcribed spacer region of *M. larici-populina* as described in Eberl et al. (2018). The pathogen was amplified by infecting 1-year-old trees, and after 2–3 weeks uredospores were harvested with a scalpel and a brush. Spores were stored at -20°C until the start of the experiment either dried over silica overnight (photosynthesis experiment and transcriptome experiment) or used within 2 months after harvesting (isoprene experiment). Plants were inoculated with the fungus by spraying a mixture of water and spores (dry: 1 mg ml^-1^ and fresh: 1.5 mg ml^-1^) on the abaxial side of each leaf (approximately 1 ml per leaf) and covering each tree with a polyethylene terephthalate (PET) bag (Bratschlauch, Toppits, Minden, Germany), which was kept closed for 1 day to ensure sufficient humidity for spore germination. Control groups received the same treatments but were sprayed with water only. First sporangia in the rust-infected trees were visible on the abaxial side of the leaves at 7 dpi (days post-infection) (Supplementary Figure S1), which matches the time course of infection in the literature (Hacquard et al., 2011). Under natural conditions individual poplar leaves are usually exposed to many cycles of rust infection. We decided to investigate only a single cycle of infection to better determine which stage of infection influences plant processes.

### Photosynthesis Measurements

Photosynthetic parameters were measured on the second mature leaf (counting from the apex) of six trees from both groups (“control” and “rust-infected”; *n* = 6) at six different time points: 1 day before rust infection (-1 dpi), 4 h post-infection (hpi), 1, 7, and 10 dpi. Due to unexpectedly high CO_2_-levels in the air supply (Supplementary Figure S3) at one measurement time (4 dpi), we excluded this data point from further analysis. All measurements were conducted at the same time of the day (9 am–12 pm) except for 4 hpi (1 pm–4 pm). The leaf was put into a custom-made single leaf chamber [chamber: polyoxymethylene, lid: poly(methyl methacrylate), openings sealed with sponge rubber; for picture see Supplementary Figure S2], which was connected to a LI-6400XT Portable Photosynthesis System (LI-COR, Lincoln, NE, United States). For each treatment a separate chamber was used to avoid contamination by fungal spores of the control leaves. The air supply for the LI-6400XT was filtered through active charcoal and humidified to 12%. An LED lamp (850 PAR at leaf position; 5 W, warm white and cool white; Roschwege GmbH, Greifenstein, Germany; for spectrum see Supplementary Figure S4) was placed over the leaf chamber as light source. The two different treatments were measured alternatingly to avoid temporal effects for one of the groups. The leaf was allowed to equilibrate to the LED light for 10 min before it was connected to the LI-6400XT, and photosynthetic parameters (photosynthetic rate, stomatal conductance and intercellular CO_2_) were measured for 10 min. The photosynthetic rate and stomatal conductance were normalized to the leaf area, which was determined with Photoshop CS5 (Adobe, San Jose, CA, United States) from a picture taken at the end of the experiment with a reference field of known size. As the trees were not actively growing during the experiment, the leaf size was assumed to be the same throughout the whole experiment. After the last measurement, the leaf was flash-frozen in liquid nitrogen for phytohormone and sugar analysis.

### Chemical Analyses

Phytohormones and sugars were analyzed from 10 mg freeze-dried, ground leaf material of the photosynthesis experiment (*n* = 6). Phytohormone analysis was carried out on an LC/MS/MS system as previously described (Eberl et al., 2018). Data were processed using ANALYST 1.5.2 (AB Sciex, Framingham, MA, United States) and hormones were quantified relative to the peak area of their corresponding standard (D_4_-salicylic acid and D_6_-abscisic acid; Santa Cruz Biotechnology, Dallas, TX, United States). For sugar analysis, extracts were diluted 1:10 with water prior to analysis on an Agilent 1200 HPLC system (Agilent, Santa Clara, CA, United States) coupled to an API 3200 tandem mass spectrometer (AB Sciex). The analytes were separated on an hydrophobic interaction liquid chromatography (HILIC)-column (apHera NH_2_ Polymer; 15 × 4.6 mm, 5 μm; Supelco, Bellefonte, PA, United States) with a water/acetonitrile gradient (flow, 1.0 ml min^-1^), for more details see Madsen et al. (2015). The data were processed using ANALYST 1.5.2 (AB Sciex) and the compounds were quantified using an external standard curve. For this, a mixture of glucose, fructose and sucrose (Sigma-Aldrich, St. Louis, MO, United States) was analyzed at six different concentrations ranging from 20 to 1.25 μg ml^-1^.

### Isoprene Measurements

Isoprene emission from rust-infected and control trees was measured from the second mature leaf from the apex in six trees of each treatment (*n* = 6). The same conditions, setup and time points (from 1 to 10 dpi) as for the photosynthesis experiment were used, but the material of the single leaf chamber was changed to aluminum (Supplementary Figure S2) to avoid volatile contamination, while the lid was still made from poly(methyl methacrylate). The ambient temperature of 20°C used in our experiments is lower than the temperature applied in comparable studies on isoprene emission (e.g., Brilli et al., 2007) but better simulates the conditions that trees of this poplar species and the pathogen experience in the field. Furthermore, the relative humidity was reduced to 6% in order to avoid excessive transpiration inside the chamber (due to a larger leaf size compared to the photosynthesis experiment). Isoprene emission was analyzed with a proton transfer reaction mass spectrometer (PTR-MS; Ionicon Analytik, Innsbruck, Austria). A detailed description of the PTR-MS can be found in Lindinger and Jordan (1998). Before starting the analysis the PTR-MS was calibrated by using the Gas Calibration Unit, a system generating clean air mixed with precise flows of an isoprene gas standard (Ionicon Analytik). The capillary line of the PTR-MS was connected with the outflow of the leaf chamber. The proton transfer reactions occurred in the reaction chamber (drift tube) between the primary ion H_3_O^+^ coming from the ion source, and the isoprene in the sampled air. In the drift tube the pressure was in the range of 2.3 mbar and the E/N ratio (electric field/particle density) was 137 Td (1 Td = 10^-17^ V cm^2^). Isoprene was monitored at the mass signal 69 (*m/z*). The raw count-rate signal intensity () of the isoprene was normalized (ncps) to the cps sum of the primary ion and water cluster, and to the drift tube pressure. Each leaf was given a 10 min equilibration period in the leaf chamber under LED light. Then the leaf chamber was connected to the PTR-MS to monitor isoprene emission for 15 min. This time was enough to reach steady-state conditions of isoprene emission. The average of cps during the steady-state period was used to calculate the emission rate, from which the background (empty leaf chamber, Supplementary Figure S2) was subtracted. Isoprene emission was normalized to the leaf area, which was determined by a picture taken at the beginning of the experiment. As the trees were not actively growing during the experiment, the leaf size was assumed to be the same throughout the whole experiment. Leaf area was calculated as described in “Photosynthesis measurements.” After the last measurement (10 dpi), the second mature leaf was flash-frozen immediately in liquid nitrogen for metabolite analysis.

### Analysis of MEP Pathway Metabolites

Leaves sampled from the isoprene experiment (*n* = 6) were ground in liquid nitrogen and then lyophilized. The MEP pathway metabolites (see Figure 5 legend) were extracted twice with a 250 μl solution of 50% acetonitrile containing 10 mM ammonium acetate, pH 9.0, using 5 mg dry tissue. After vortexing and micro-centrifugation, 200 μl of the supernatant from both extracts were combined, transferred into a new 1.5 ml tube and dried under a stream of nitrogen gas at 40°C. The residue was dissolved in 100 μl of 10 mM ammonium acetate, pH 9.0, and, after vortexing, 100 μl of chloroform was added. The upper aqueous phase, separated by centrifugation, was transferred into a new tube and diluted with 1 volume of acetonitrile. After centrifugation for 5 min to remove any precipitate, the supernatant was transferred to an HPLC vial. MEP pathway metabolites were analyzed on an Agilent 1260 Infinity HPLC system (Agilent) connected to an API 5000 triple quadrupole mass spectrometer (AB Sciex). A 5 μl portion of the extract was injected and the metabolites were separated on a HILIC XBridge Amide column (150 × 2.1 mm, 3.5 μm; Waters, Milford, MA, United States) with a HILIC guard column containing the same sorbent (3.5 μm, 10 × 2.1 mm) and a SSITM high pressure pre-column filter (Sigma-Aldrich) using two solvents: 20 mM ammonium bicarbonate adjusted to pH 10.5 with ammonium hydroxide (solvent A) and 80% acetonitrile containing 20 mM ammonium bicarbonate, pH 10.5 (solvent B). The solvent gradient profile started with 100% of solvent B which decreased to 60% in the first 15 min, followed by an isocratic gradient with solvent B. Separation was performed at 25°C with a flow rate of 500 μl min^-1^. The mass spectrometer operated in negative ionization mode with ion spray voltage -4500 eV, turbo gas temperature 700°C and nebulizer gas 70 psi. The MEP pathway metabolites were analyzed using the MRM conditions described by Wright et al. (2014). The metabolite concentrations were calculated by using external standard curves, and were normalized to the [^13^C]-labeled internal standards of each intermediate (González-Cabanelas et al., 2016) added to the extract after the first extraction step.

### Leaf Pigment Analysis

Leaves sampled from the isoprene experiment at 10 dpi were ground in liquid nitrogen and 50 mg of fresh tissue was extracted in light-protected tubes with 1 ml of acetone by shaking for 6 h at 4°C in the dark. After centrifugation for 5 min at 2350 g at 4°C, 800 μl of the extract was transferred into a new light-protected tube and 200 μl of water was added. After spinning the samples for 1 min at 5000 rpm at 4°C, they were transferred to brown glass vials for analysis on an Agilent 1100 Series HPLC with UV/VIS diode array detector. The detector was set at 445 nm for the detection of carotenoids and at 650 nm for the chlorophylls. The pigments were separated on a Supelcosil column LC-18 (7.5 cm × 4.6 mm × 3 μm; Sigma-Aldrich) using an acetone (solvent A)/1 mM NaHCO_3_ (in water, solvent B) gradient with a flow rate of 1.5 ml min^-1^. The initial mobile phase consisted of 65/35% (v/v) solvent A/solvent B. Then, solvent A was linearly increased to 90% within 12 min and to 100% over 8 min. 100% solvent A was kept for 2 min and then decreased to 65% again within 3 min. Quantification was done using external standard curves. Authentic standards of the chlorophylls and β-carotene (Santa Cruz Biotechnology) were analyzed in a range from 0.1 to 0.00625 mg ml^-1^. Lutein, neoxanthin, and violaxanthin were assumed to have the same response factor as β-carotene.

### Transcriptome Analysis

To investigate transcriptional changes in black poplar leaves upon rust infection, eight trees grown from green cuttings were selected and half of them were inoculated with *M. larici-populina* uredospores. Leaves from the trees of both treatments (“control” and “rust-infected”; *n* = 4) were harvested 8 dpi and flash-frozen in liquid nitrogen. RNA was isolated with the InviTrap Spin Plant Mini Kit (Stratec Biomedical AG, Birkenfeld, Germany), including a DNase digestion (DNase kit, Qiagen, Hilden, Germany). RNA concentration and quality was analyzed with a NanoDrop 2000c spectrophotometer (Peqlab Biotechnologie GmbH, Erlangen, Germany) and the RNA 6000 Nano Kit on a Bioanalyzer (Agilent). Sequencing was done at the Max Planck-Genome-Centre (Köln, Germany) on a HiSeq 2500 (Illumina, San Diego, CA, United States) with 9 Mio reads per sample. Quality control measures, including the filtering of high-quality reads based on fastq file scores, the removal of reads containing primer/adapter sequences, and trimming of the read length, were carried out using CLC Genomics Workbench v9.1^3^. The same software was used for *de novo* transcriptome assembly, combining two replicates of each RNA-Seq treatment group, and selecting the presumed optimal consensus transcriptome as previously described (Vogel et al., 2014). The final *de novo* reference transcriptome assembly (backbone) of *P. nigra* contained 81,580 contigs (sets of overlapping sequence segments that together represent a continuous region of the original RNA). Minimum contig size was 250 bp with an N50 contig size of 1320 bp. The transcriptome was annotated using BLAST, Gene Ontology (GO) and InterPro terms (InterProScan, EBI), enzyme classification (EC) codes, and metabolic pathways (Kyoto Encyclopedia of Genes and Genomes, KEGG) as implemented in BLAST2GO v4.1^4^. Based on the BLAST hits, the contigs were designated as being of either plant or fungal (i.e., *M. larici-populina*) origin. To assess transcriptome completeness, we performed a BUSCO^5^ (Benchmarking Universal Single-Copy Orthologs) analysis by comparing our assembled (plant-derived only) transcript set against a set of highly conserved single-copy orthologs. This was accomplished using the BUSCO v3 pipeline (Waterhouse et al., 2017) compared to the predefined set of 303 Eukaryota single-copy orthologs from the OrthoDB v9.1 database. Our assembled transcriptome was determined to be 87.8% complete and only 3.6% of the BUSCO genes were missing. Digital gene expression analysis was carried out using CLC Genomics Workbench v9.1 to generate BAM (mapping) files, and QSeq Software (DNAStar Inc., Madison, WI, United States) was then used to estimate expression levels. The log_2_ (RPKM) values (normalized mapped read values; geometric means of the biological replicate samples) were subsequently used to calculate fold-change values. To identify differentially expressed genes, we used the Student’s *t*-test (as implemented in Qseq) corrected for multiple testing using the Benjamini–Hochberg procedure to check the false discovery rate (FDR). A gene was considered significantly differentially expressed if the FDR-corrected *p*-value was less than 0.05. Fisher’s exact test was used as part of BLAST2GO to identify the overrepresentation of GO terms among lists of differentially expressed genes between treatment groups. The GO-enriched bar charts were simplified to display only the most specific GO terms by removing parent terms representing existing child terms using the function “Reduce to most specific terms” in BLAST2GO. A GO term was considered significantly enriched if the *p*-value corrected by FDR control was less than 0.05.

### Statistics

All data were tested for statistical assumptions, i.e., normal distribution and homogeneity of variances. Whenever necessary, the data were log-transformed (salicylic acid, DMADP + IDP). For the photosynthetic parameters and the isoprene emission a two-way repeated measures ANOVA was performed using “time” as a within-subject factor and “rust infection” as a between-subject factor. For end-point measurements (phytohormones, sugars, MEP pathway metabolites and carotenoids) an independent student’s *t*-test was performed for each compound. Correlations between photosynthetic parameters and phytohormones were tested using bivariate Pearson’s *r* correlation. All statistical analyses were performed with SPSS 17.0 (SPSS, Chicago, IL, United States).

## Results

### Photosynthesis Decreases After Rust Infection but Sugar Levels Are Unaffected

In order to investigate the influence of fungal infection on photosynthesis in black poplar, we measured photosynthetic parameters at various times during the development of rust infection in the leaves.

**FIGURE 1:**
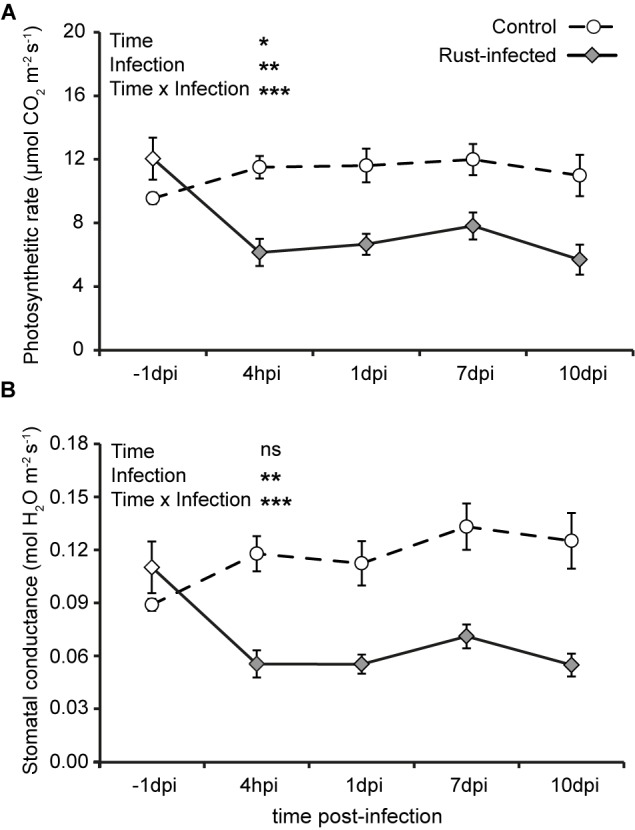
Photosynthetic parameters of rust-infected black poplar trees (filled symbols) and uninfected controls (open symbols) at different time points after infection (dpi = days post-infection, hpi = hours post-infection; –1 dpi = 1 day before infection). Measurements were made on the second mature leaf counting from the apex. Shown are means ± SEM (*n* = 6) for **(A)** photosynthetic rate (μmol CO_2_ m^-2^ s^-1^) and **(B)** stomatal conductance (mol H_2_O m^-2^ s^-1^). Results from repeated measures ANOVA using the factors “Time” (time points before and post-infection), “Infection” (rust infection), and the interaction “Time × Infection” are given as insets in the graphs (ns = not significant; ^∗^*P* < 0.05; ^∗∗^*P* < 0.01; ^∗∗∗^*P* < 0.001).

The photosynthetic assimilation rate in uninfected control trees was generally stable at 9–12 μmol CO_2_ m^-2^ s^-1^ during the time course of measurements (Figure 1A). The photosynthetic rate in rust-infected poplar trees, however, dropped by approximately 50% within the first 4 hpi from 12.1 ± 1.32 to 6.1 ± 0.86 μmol m^-2^ s^-1^. This reduction persisted throughout the later time points. The factor “rust infection” strongly influenced the photosynthetic rate, as also shown statistically [repeated measures ANOVA: *F*_(1,10)_ = 15.524, *P* = 0.003]. However, there was also a significant interaction of time and rust infection [repeated measures ANOVA: *F*_(4,40)_ = 8.891, *P* < 0.001]. The patterns of stomatal conductance were similar to those observed for photosynthetic assimilation rate. Control plants without rust infection showed a slight increase in stomatal conductance throughout the experiment from 0.09 ± 0.003 mol m^-2^ s^-1^ at the first measurement to 0.13 ± 0.012 mol m^-2^ s^-1^ during the last three measurements (Figure 1B). Rust fungus infection decreased stomatal conductance in poplar trees by more than 50% as early as 4 hpi, when it declined from 0.11 ± 0.015 to 0.06 ± 0.005 mol s^-1^ m^-2^. As the infection progressed, the stomatal conductance of rust-infected trees remained at around 50% of that measured from uninfected controls. The effect of “rust infection” was statistically highly significant [repeated measures ANOVA: *F*_(1,10)_ = 19.815, *P* = 0.001], as was the interaction of time and rust infection [repeated measures ANOVA: *F*_(4, 40)_ = 10.665, *P* < 0.001]. However, the intercellular CO_2_ levels did not differ between the different time points, nor between the two treatments, i.e., rust-infected and control black poplar leaves (Supplementary Figure S5).

After observing the reduction in photosynthetic assimilation rate in rust-infected poplar leaves, we analyzed glucose, fructose and sucrose levels in leaves harvested at 10 dpi. For these sugars, no significant changes in concentration were observed after infection of leaves by the rust fungus (Table 1).

**Table 1:** Soluble sugar levels in control and rust-infected black poplar trees 10 days post-infection expressed in mg g^-1^ dry weight.

	Control	Rust-infected	*t*	*P*
Glucose	1.31 ± 0.19	1.36 ± 0.17	-0.204	0.843
Fructose	2.02 ± 0.51	2.03 ± 0.36	-0.012	0.991
Sucrose	35.53 ± 3.76	41.44 ± 3.09	-1.213	0.253


*Measurements were made on the second mature leaf counting from the apex. Shown is mean ± SEM (*n* = 6) and *t*- and *P*-value of Student’s *t*-test.*

Our results show that photosynthetic activity is downregulated in poplar leaves right after the onset of infection with the rust fungus, and remains lower over the course of infection without affecting soluble sugars.

### Salicylic Acid (SA) and Abscisic Acid (ABA) Increase in Rust-Infected Leaves

To analyze phytohormones that might be involved in regulating anti-pathogen defense and photosynthesis, leaves were collected at 10 dpi.

**FIGURE 2:**
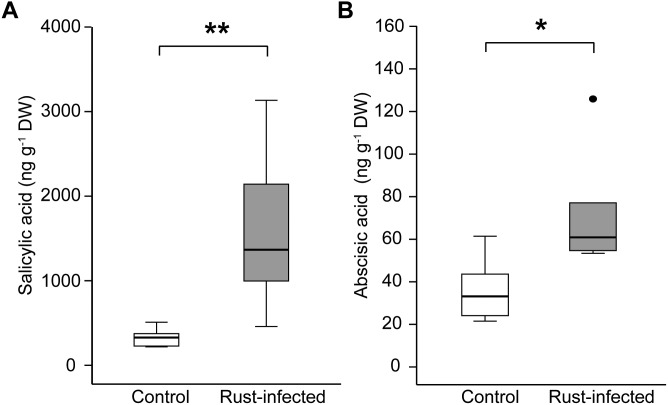
Levels of the phytohormones **(A)** salicylic acid and **(B)** abscisic acid in leaves from rust-infected black poplar trees (filled symbols) and uninfected controls (open symbols) 10 days post-infection. Measurements were made on the second mature leaf counting from the apex. Boxplots show the median with upper and lower quartile (*n* = 6) and dots for outliers; asterisks indicate statistically significant differences between groups (independent Student’s *t*-test, ^∗^*P* < 0.05, ^∗∗^*P* < 0.01).

Salicylic acid increased fivefold in rust-infected black poplar leaves compared to uninfected controls [331 ± 45 ng g^-1^ DW in controls; 1580 ± 390 ng g^-1^ DW in rust-infected trees, Figure 2A], which is highly significant [Student’s *t*-test, *t*_(10)_ = -4.687, *P* = 0.001]. ABA, a phytohormone regulating stomatal opening, among other processes, also increased in rust-infected black poplar leaves, reaching a level twice as high as in the control leaves (36.2 ± 6.10 ng g^-1^ DW in controls; 72.1 ± 11.34 ng g^-1^ DW in rust-infected trees, Figure 2B). Additionally, ABA levels in rust-infected leaves correlated negatively with stomatal conductance and photosynthetic rate measured at 10 dpi (Table 2). In control leaves, however, no such correlation could be observed.

**Table 2:** Correlation between phytohormone levels and photosynthetic parameters of control and rust-infected black poplar trees 10 days post-infection (data from Figures 1, 2).

	Control		Rust-infected	
		
	Photosynthetic rate	Stomatal conductance	Photosynthetic rate	Stomatal conductance
Salicylic acid	*ρ* = -0.384	*ρ* = -0.589	*ρ* = -0.537	*ρ* = -0.661
Abscisic acid	*ρ* = -0.257	*ρ* = -0.186	***ρ* = -0.987^∗∗∗^**	***ρ* = -0.969^∗∗^**


*Measurements were made on the second mature leaf counting from the apex. *ρ*, Pearson correlation coefficient. Significant correlations are highlighted in bold and marked with asterisks (^∗∗∗^*P* < 0.001; ^∗∗^*P* < 0.01; *n* = 6).*

Taken together, we could show that rust infection increased SA as well as ABA in poplar leaves, whereas the latter shows a negative relation to photosynthetic parameters.

### Rust Infection Does Not Affect Isoprene Emission

To evaluate the effect of pathogen infection on isoprene emission, we monitored leaves of infected and uninfected young poplar trees using a PTR-MS at different time points before and during infection with the rust fungus (*M. larici-populina*).

**FIGURE 3:**
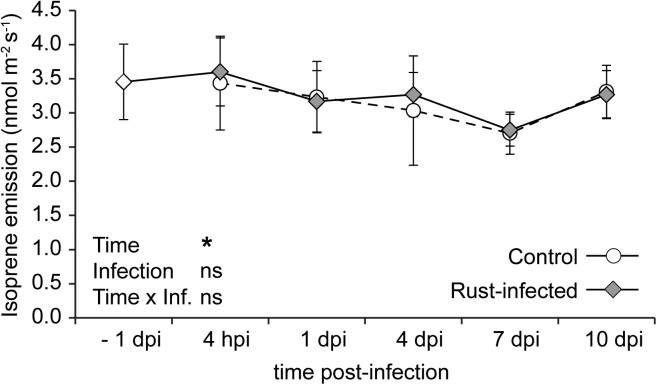
Isoprene emission from leaves of rust-infected black poplar trees (filled symbols) and uninfected controls (open symbols) at different time points after infection (dpi = days post-infection, hpi = hours post-infection; –1 dpi = 1 day before infection). Measurements were made by proton-transfer reaction mass spectrometry (PTR-MS) on the second mature leaf counting from the apex. Shown are means ± SEM (*n* = 6). Results from repeated measures ANOVA using the factors “Time” (time points before and post-infection), “Infection” (rust infection) and the interaction “Time × Infection” are given in the graph (ns = not significant; ^∗^*P* < 0.05).

The isoprene emission from uninfected controls showed slight variations throughout the time course of the experiment, ranging from 2.7 to 3.4 nmol m^-2^ s^-1^. Infection with the rust did not significantly change emission of isoprene compared to control trees or compared to the levels measured before the pathogen inoculation (Figure 3). The fluctuations over time were similar to those in uninfected controls, leading to a significant statistical effect of “time” [repeated measures ANOVA: *F*_(4,40)_ = 1.475, *P* = 0.031]. However, there was no significant effect of “infection” or the interaction “time × infection” on isoprene emission. This shows that rust infection does not affect the emission of isoprene from black poplar trees.

### Rust Infection Did Not Influence the Genes, Intermediates or Most Products of the MEP Pathway

**FIGURE 4:**
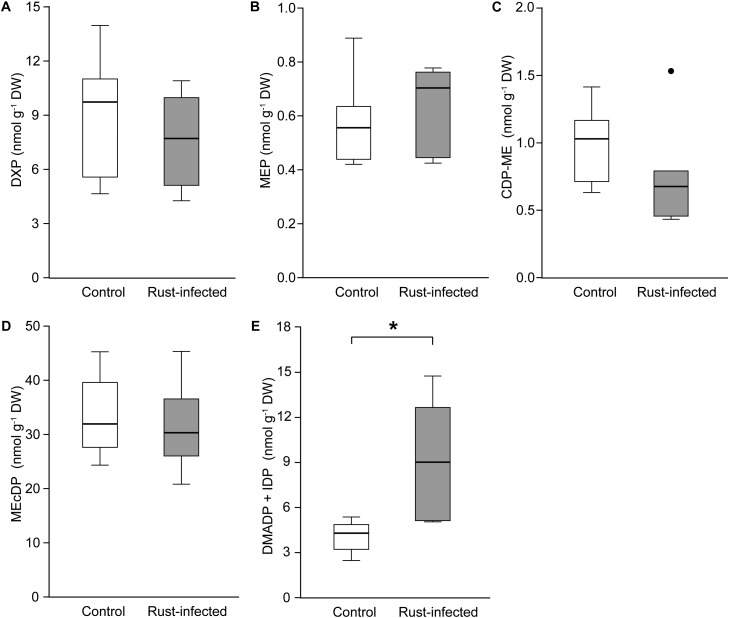
Concentrations of intermediates of the methylerythritol-4-phosphate (MEP) pathway in leaves of rust-infected black poplar trees (filled bars) and uninfected controls (open bars) 10 days post-infection. Boxplots show the median with upper and lower quartile as bars (*n* = 6) and dots for outliers; asterisks indicate statistically significant differences between groups (independent Student’s *t*-test, ^∗^*P* < 0.05). Intermediates are shown in the order occurring in the pathway: **(A)** 1-deoxy-D-xylulose-5-phosphate (DXP); **(B)** 2*C*-methyl-D-erythritol-4-phosphate (MEP); **(C)** 4-diphosphocytidyl-2*C*-methyl-D-erythritol (CDP-ME); **(D)** 2*C*-methyl-D-erythritol-2,4-cyclodiphosphate (MEcDP); **(E)** dimethylallyl diphosphate and isopentenyl diphosphate (DMADP + IDP, not separable in our analysis).

We analyzed intermediates of the MEP pathway and levels of the photosynthetic pigments, chlorophylls and carotenoids. Like isoprene, these are also produced from DMADP and IDP originating from the MEP pathway. The analyzed leaves were sampled together with any fungal spores and mycelium present in infected leaves. The MEP pathway intermediates, DXP, MEP, CDP-ME, and MEcDP, were present at similar levels in rust-infected and control leaves (Figures 4A–D). However, the amount of DMADP and IDP (DMADP + IDP), the final products of both the MEP and MVA pathways (not separable in our LC-MS analysis), were significantly higher in rust-infected leaves [Student’s *t*-test, *t*_(10)_ = -3.503, *P* = 0.006] (Figure 4E). The concentration of DMADP + IDP in rust-infected leaves was more than double that of control leaves (4.1 ± 0.46 nmol g^-1^ FW in controls; 9.3 ± 1.60 nmol g^-1^ FW in rust-infected trees). On the other hand, many of the major isoprenoids that are known to be produced from MEP pathway-derived C_5_ units, including the carotenoids, lutein, neoxanthin, violaxanthin, and chlorophylls a and b, did not change after rust infection (Table 3). β-Carotene, however, increased by more than 50% in leaves from rust-infected poplars compared to uninfected controls (0.59 ± 0.072 mg g^-1^ FW in controls; 0.93 ± 0.090 mg g^-1^ FW in rust-infected; Student’s *t*-test, *t*_(10)_ = -3.100, *P* = 0.011). The orange-colored uredospores of the rust fungus *M. larici-populina* were also analyzed separately to determine if these contained any carotenoids, and β-carotene, but none of the other poplar carotenoids, was found (Supplementary Table S1). The concentration of β-carotene was more than threefold higher in fungal spores than in uninfected poplar leaves.

**Table 3:** Carotenoid and chlorophyll levels in control and rust-infected black poplar trees 10 days post-infection expressed in mg g^-1^ fresh weight.

	Control	Rust-infected	*t*	*P*
β-Carotene	0.59 ± 0.07	0.93 ± 0.09	-3.100	**0.011**
Lutein	1.43 ± 0.05	1.35 ± 0.06	0.978	0.351
Neoxanthin	0.31 ± 0.01	0.29 ± 0.01	1.572	0.147
Violaxanthin	0.28 ± 0.03	0.32 ± 0.02	-1.204	0.256
Chlorophyll *a*	1.01 ± 0.05	0.92 ± 0.01	1.762	0.133
Chlorophyll *b*	0.68 ± 0.03	0.63 ± 0.01	1.467	0.192


*Measurements were made on the second mature leaf counting from the apex. Shown is mean ± SEM (*n* = 5–6) and *t*- and *P*-value of Student’s *t*-test. Bold numbers indicate significant differences.*

**FIGURE 5:**
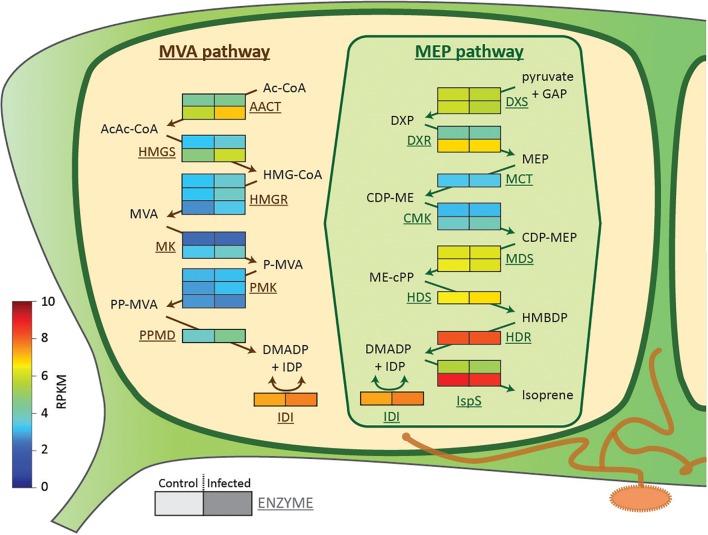
Expression of biosynthetic genes of the two isoprenoid pathways in black poplar leaves from rust-infected trees (“Infected,” right box) compared to uninfected controls (“Control,” left box). Shown are RPKM (reads per kilobase of transcript per million mapped reads) values of contigs annotated as enzymes of the MVA and MEP pathways (*n* = 4 biological replicates). Each row with two boxes (Control and Infected) represents one contig. **Metabolites** (in black): acetyl coenzyme A (CoA) (Ac-CoA), acetoacetyl coenzyme A (AcAc-CoA), 3-hydroxy-3-methylglutaryl coenzyme A (HMG-CoA), mevalonate (MVA), mevalonate-5-phosphate (P-MVA), mevalonate-5-diphosphate (PP-MVA), dimethylallyl diphosphate (DMADP), isopentenyl diphosphate (IDP), D-glyceraldehyde-3-phosphate (GAP), 1-deoxy-D-xylulose-5-phosphate (DXP), 2*C*-methyl-D-erythritol-4-phosphate (MEP), 4-diphosphocytidyl-2*C*-methyl-D-erythritol (CDP-ME), 4-diphosphocytidyl-2*C*-methyl-D-erythritol-2-phosphate (CDP-MEP), 2*C*-methyl-D-erythritol-2,4-cyclodiphosphate (MEcDP), and 1-hydroxy-2-methyl-2-(*E*)-butenyl-4-diphosphate (HMBDP). **Enzymes** (in brown/green): AcAc-CoA thiolase (AACT), HMG-CoA synthase (HMGS), HMG-CoA reductase (HMGR), mevalonate kinase (MK), phosphomevalonate kinase (PMK), diphosphomevalonate decarboxylase (PPMD), isopentenyl-diphosphate isomerase (IDI), DXP reductoisomerase (DXR), MEP cytidyltransferase (MCT), CDP-ME kinase (CMK), MEcDP synthase (MDS), HMBDP synthase (HDS), and HMBDP reductase (HDR).

DMADP and IDP, the C_5_ building blocks of all isoprenoids, can be produced by either the plastidic MEP pathway or the cytosolic MVA pathway (Rodríguez-Concepción and Boronat, 2015). To determine whether rust infection could influence either pathway, we carried out transcriptome analysis of uninfected and rust-infected black poplar trees. No differential expression of genes involved in the MEP pathway was observed (Figure 5). However, genes encoding biosynthetic enzymes of the MVA pathway increased in expression in rust-infected leaves compared to uninfected controls (Figure 5). Two enzymes catalyzing early steps of the MVA pathway, acetoacetyl-coenzyme A thiolase and 3-hydroxy-3-methylglutaryl-coenzyme A (HMG-CoA) synthase, had especially higher RPKM values (reads per kilobase of transcript per million mapped reads) in leaves infected with the rust fungus. The transcriptome analysis also identified MVA pathway transcripts of fungal origin, likely from *M. larici-populina*. Contigs annotated as fungal HMG-CoA synthase and all downstream MVA pathway enzymes were detected in the transcriptome of rust-infected leaves (Supplementary Table S2), but not in control leaves.

Thus rust infection did not affect the levels of intermediates of the MEP pathway. However, the amounts of the isoprenoid building blocks DMADP + IDP increased, as a result of up-regulation of the MVA pathway. In addition, there was an increase in β-carotene, which likely arose from the isoprenoid biosynthesis by the rust fungus itself.

## Discussion

*Melampsora* rusts are among the most devastating pathogens of poplar trees (Pei and Shang, 2005). We investigated the effect of rust infection (*M. larici-populina*) on photosynthesis and the emission of isoprene in black poplar (*P. nigra*).

Our results indicate that black poplar actively downregulates photosynthesis when it is subjected to rust infection. Yet despite the spatial and metabolic connections between photosynthesis and isoprene formation, the emission of isoprene was completely unaffected by the presence of the rust fungus. Consistent with this, fungal infection also did not change the expression of biosynthetic genes or levels of intermediates of the MEP pathway, the route to formation of the C_5_ units used in isoprene biosynthesis. However, an increase in the quantity of DMADP and IDP was observed, which may be attributed to the increased activity of the MVA pathway (the alternative route to producing C_5_ isoprenoid units) in poplar or the fungus itself.

### Rust Infection Drastically Reduces Black Poplar Photosynthesis

Despite the central importance of photosynthesis in supplying plants with carbon, energy, and reducing equivalents, we observed an immediate and sustained decrease in photosynthetic activity in rust-infected black poplar leaves of nearly 50%, from 4 hpi to 10 dpi (Figure 1). Infection of poplar with *Melampsora* leaf rust was earlier reported to be associated with a marked reduction in photosynthesis (Zhang et al., 2010, 2016; McKown et al., 2014; Jiang et al., 2016; Gortari et al., 2018), but the detailed temporal dynamics of this reduction had not been studied. In willow, a similar pattern of decreased net photosynthetic rate was observed after rust infection, but stomatal conductance changed only at late time points (Toome et al., 2010). A negative impact of rust on transcripts of photosynthetically relevant genes in poplar was also reported at 6 dpi (summarized in Major et al., 2010). Interestingly, we did not observe any changes in the intercellular CO_2_ concentration (*C*_i_) after rust infection (Supplementary Figure S5). A decreased *C*_i_ would be expected when the assimilation rate decreases after stomatal closure due to the lowered availability of CO_2_. The lack of decrease of *C*_i_ in our study might be explained by temporal dynamics, i.e., a fast reduction and subsequent equilibration of *C*_i_ before the first measurement. Alternatively, a signal – most likely phytohormonal – might have regulated stomatal conductance and assimilation rate simultaneously, so that reduced uptake and reduced consumption of CO_2_ would balance out without any net effect on the *C*_i_. A decline in stomatal conductance and assimilation rate with stable *C*_i_ has been observed before after herbivory (Meza-Canales et al., 2017). Also, the influence of biotic stress-related phytohormones on photosynthesis has been shown in other systems (Popova et al., 1988; Tang et al., 2017). A transcriptional analysis conducted at early time points of infection would help to better understand the physiological mechanisms leading to the rapid decline in photosynthesis.

In addition to the decrease in photosynthesis we observed elevated levels of SA and ABA (Figure 2) in poplar leaves upon rust infection. SA is known to play a central role in plant defense against biotrophic organisms such as rusts or mildew, and induces a hypersensitive response, the expression of pathogenesis-related genes and other responses (Derksen et al., 2013). ABA, on the other hand, is primarily known to mediate responses to abiotic stresses such as drought (Tuteja, 2007) and to control stomatal closure (Acharya and Assmann, 2009). Stomata are natural openings through which the rust fungus can enter the intercellular spaces of the leaf within the first 6 h after inoculation (Hacquard et al., 2011). Yet rapid closure of stomata might prevent the pathogen from entering, as was observed in tomato leaves infected by *Pseudomonas syringae* (Melotto et al., 2006). A similar mechanism might occur in poplar leaves and would explain the fast decrease in stomatal conductance and the increased levels of ABA observed in infected tissue. Phytohormone analysis of earlier time points and exogenous application of ABA and SA will help to disentangle the signaling networks between these two hormones and control of photosynthesis. Considering the fast response of photosynthetic parameters to rust infection and the changes in phytohormone content, we infer an active control of stomatal closure by the plant. Previous work suggested that a mechanical disturbance by fungal hyphae could also be involved in reducing stomatal conductance (Jiang et al., 2016), perhaps especially at later time points.

### Soluble Carbohydrate Levels Are Maintained in Rust-Infected Leaves

Although rust infection triggered a drastic decrease in the photosynthetic assimilation rate of poplar (Figure 1), soluble sugar content did not decrease in rust-infected compared to uninfected control leaves (Table 1). Given the rapid production of sucrose and hexose sugars as photosynthetic assimilates, it is surprising that their levels were not affected by the decline in photosynthesis. Soluble sugars can also originate from breakdown of storage carbohydrates or transport from other tissues. Since biotrophic pathogens utilize hexose sugars from their hosts (Voegele and Mendgen, 2011), it is assumed that infected tissues become carbon sinks even when photosynthetically active in order to satisfy the increased demand for carbon (Berger et al., 2007). This would require the mobilization of carbohydrates from other parts of plant. Such mobilization might account for the decline in wood production in poplar observed on rust infection, which causes significant economic losses in infected plantations (Frey et al., 2005; Wan et al., 2013). Another explanation for the maintenance of constant sugar levels in rust-infected leaves with a concurrent decrease in assimilation rate could be the reduced export of sugars. Further work is needed to elucidate the mechanisms responsible for maintaining sugar levels under these conditions.

### Isoprene Emission Is Not Affected by Rust Infection

Isoprene is emitted in large amounts by poplar and other tree species, and it is assumed to protect leaves from heat stress or reactive oxygen species (Sharkey et al., 2008). However, the role of isoprene under biotic stress is poorly studied. We observed no influence of rust infection on isoprene emission from black poplar (Figure 3). Similar patterns of stable isoprene emission were observed under drought stress conditions (Pegoraro et al., 2004; Brilli et al., 2007), which also trigger ABA-mediated stomatal closure. On the other hand, studies on the effects of insect herbivory or mechanical wounding have reported variable outcomes, showing either an increased (Brilli et al., 2011), stable (Müller et al., 2015), decreased (Brilli et al., 2009; Jardine et al., 2013; Jiang et al., 2018), or time-dependent (Loreto and Sharkey, 1993; Loreto et al., 2006; Portillo-Estrada et al., 2015) isoprene emission patterns after stress application.

In contrast to our results, another study investigating the influence of rust infection on poplar trees observed lower isoprene emissions from infected compared to uninfected trees (Jiang et al., 2016) possibly due to the more severe level of infection. Although in our study rust significantly decreased photosynthetic parameters and altered hormone levels, our infected leaves did not have necrotic lesions (Supplementary Figure S6). Such necrotic lesions, however, were present on the leaves used by Jiang et al. (2016), and may have induced the reduction in isoprene emission. Necrosis leads to premature death of cells and hence could reduce the area of living tissue with which the plant synthesizes isoprene. The stability of isoprene emission under various stress conditions suggests that this compound is of vital importance to the physiology of isoprene-emitting plants. This importance might be due to a direct effect of isoprene, for example, by reducing oxidative stress, or an indirect effect by maintaining flux through the MEP pathway (Logan et al., 2000), which provides essential compounds for plant metabolism. After many years of research, the physiological role of isoprene is still to a large extent unknown.

### Involvement of Plant and Fungal MVA Pathways in Isoprenoid Production in Infected Leaves

Considering the tight metabolic connections between photosynthesis and the MEP pathway, we expected to observe lower MEP pathway activity after rust infection due to the reduction in photosynthesis. However, the transcription of genes encoding biosynthetic enzymes of the MEP pathway did not change after rust infection (Figure 5). In addition, the stable levels of MEP pathway intermediates (Figures 4A–D) suggest a constant metabolic flux through the pathway despite fungal infection. Consistent with this, the levels of the chlorophylls and most carotenoids, the main non-volatile products of the MEP pathway, did not change after rust infection (Table 3). Constant levels of the chlorophylls in leaves after rust infection were also recently reported in another poplar species (Gortari et al., 2018). However, the levels of DMADP and IDP (quantified together in our LC-MS analysis) increased after infection (Figure 4E). DMADP and IDP can be produced by both the plastidic MEP pathway and the cytosolic MVA pathway (Hemmerlin et al., 2012). Since the cells in the leaf were disrupted for chemical analysis, the DMADP and IDP present in both cellular compartments were analyzed simultaneously. An increased transcript abundance of genes involved in the early steps of the MVA pathway (Figure 5) suggests the increased DMADP + IDP levels observed in infected tissue might be derived from this pathway. This explanation is also supported by the recently reported increased emission of sesquiterpenes upon rust infection of poplar (Eberl et al., 2018). Alternatively, the increased DMADP + IDP levels could have resulted from the fungal metabolism in rust-infected tissue. Future research using ^13^C-labeled CO_2_ (Ghirardo et al., 2014) or glucose (Hemmerlin et al., 2012) could elucidate the biosynthetic origin of the increased DMADP + IDP.

In addition to higher DMADP + IDP-levels we also found higher amounts of β-carotene in rust-infected leaves compared to controls (Table 3). When rust spores were analyzed separately for carotenoids, high concentrations of β-carotene but no other carotenoids were found (Supplementary Table S1). We then analyzed the transcriptome of infected poplar leaves for *Melampsora*-specific genes involved in isoprenoid biosynthesis and found transcripts for genes encoding all steps of the MVA pathway (Supplementary Table S2). In contrast to plants where carotenoids are produced *via* the MEP pathway, their biosynthesis in fungi occurs *via* the MVA pathway (Disch and Rohmer, 1998). Taken together, it is likely that *M larici-populina* produces β-carotene in its hyphae and spores. The fungus *M. larici-populina* in the infected tissue is therefore the most probable cause of the increased β-carotene and DMADP + IDP levels. The fungus might use this pigment in spores to attract spore dispersers (Cano et al., 2013) or in hyphae to scavenge oxygen radicals (Davoli and Weber, 2002) that are produced by plants as defenses against infection (Ferreira et al., 2006).

## Conclusion

Our study provides new insight into the impact of a widely distributed biotrophic pathogen on photosynthesis and isoprene formation in a poplar species. The hormone-mediated closure of stomata upon infection diminishes the photosynthetic activity of infected leaves and hence reduces the ability of the tree to assimilate new carbon. However, infected leaves maintain stable carbohydrate levels and continue to emit isoprene at unchanged rates despite carbon consumption by the pathogen. Most likely, infected leaves import soluble sugars from elsewhere in the tree. In the long term, rust disease may therefore result in reduced biomass production by poplar resulting in significant declines in plantation yield. However, since the level of isoprene emission was not affected, the influence on atmospheric chemistry will likely be minimal.

## Author Contributions

FE designed the experiments and wrote the article. FE and EP performed the experiments and analyzed the data except for the transcriptome. HV analyzed the transcriptome data. DV designed and constructed the experimental equipment. LW, AH, SU, and JG conceived the project and complemented writing.

## Conflict of Interest Statement

The authors declare that the research was conducted in the absence of any commercial or financial relationships that could be construed as a potential conflict of interest.

## Footnotes

1 https://ohioline.osu.edu/factsheet/plpath-tree-8

2 www.webofknowledge.com

3 http://www.clcbio.com

4 http://www.blast2go.de

5 http://busco.ezlab.org
