# Supplementary material for: Rust Infection of Black Poplar Trees Reduces Photosynthesis but Does Not Affect Isoprene Biosynthesis or Emission
Source: Front Plant Sci. 2018 Nov 27;9:1733. doi: 10.3389/fpls.2018.01733 (PMC6277707; doi:10.3389/fpls.2018.01733)

Supplementary Material

Rust infection of black poplar trees reduces photosynthesis but does not affect isoprene biosynthesis or emission

**Franziska Eberl, Erica Perreca, Heiko Vogel, Louwrance Wright, Almuth Hammerbacher, Daniel Veit, Jonathan Gershenzon, Sybille B. Unsicker^*^**

*** Correspondence:** Dr. Sybille Unsicker: [sunsicker@ice.mpg.de](mailto:sunsicker@ice.mpg.de)

**
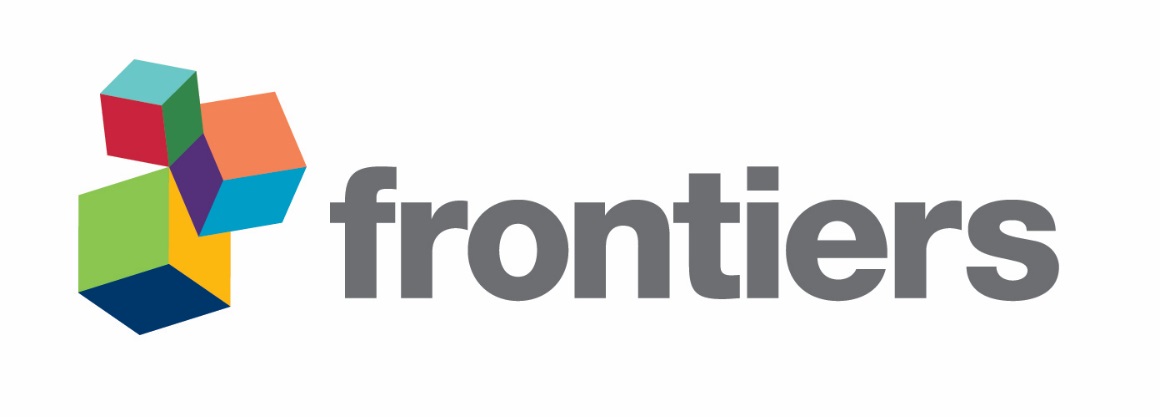
**

# Supplementary Figures and Tables

## Supplementary Figures

**
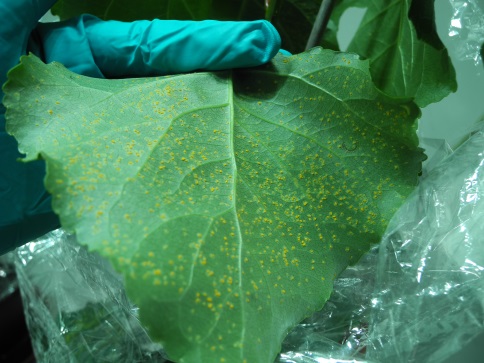

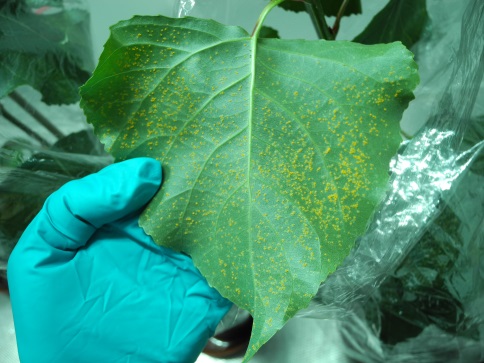

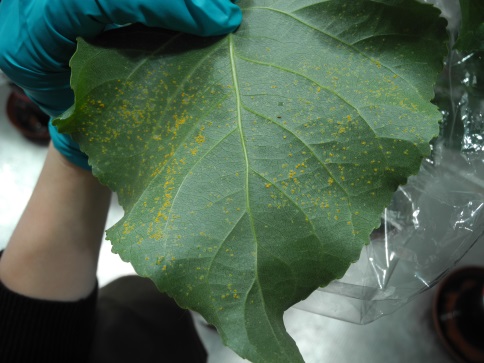

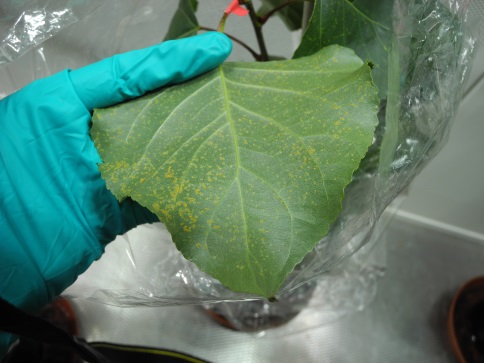

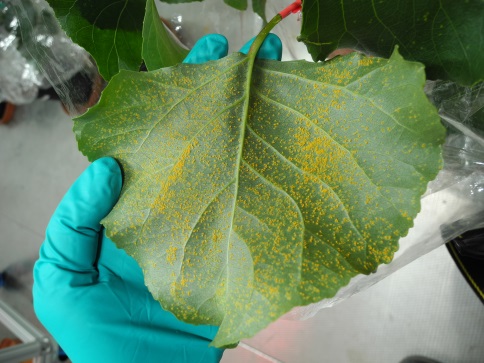

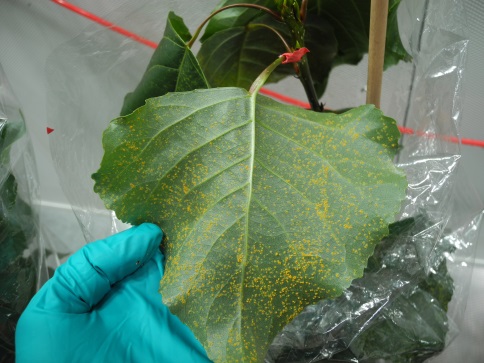
**

**Supplementary Figure S1.** Second mature leaf from all rust-infected black poplar trees used in the isoprene experiment at 7 dpi, the first occurrence of rust sporangia. Pictures were taken from the abaxial side of the leaves.

**
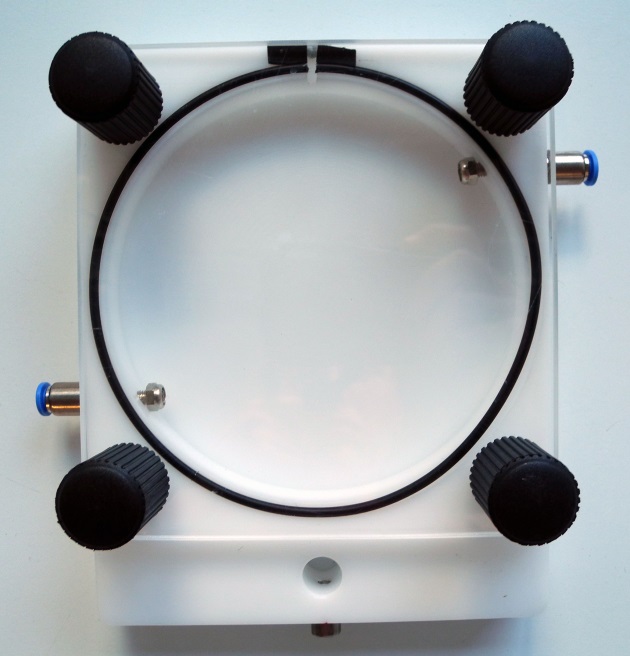

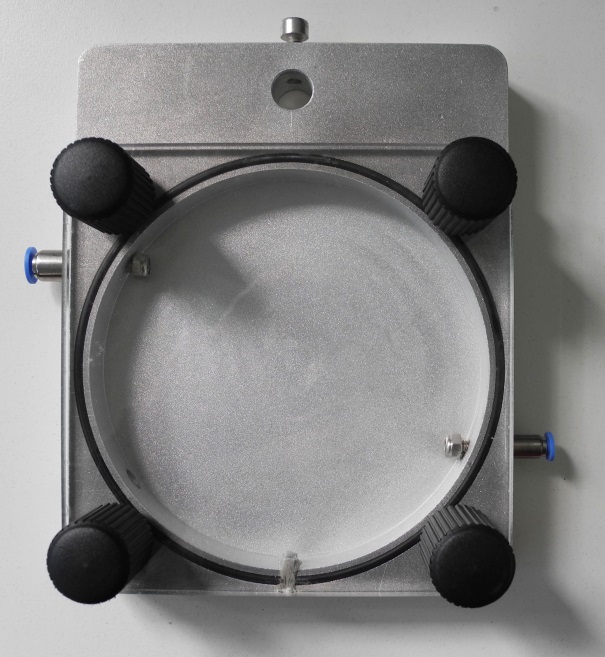
**

**B**

**A**

**Supplementary Figure S2**. Custom-made single leaf chambers (diameter 13 cm) for photosynthesis experiment (A) and isoprene experiment (B). Air enters through Teflon tubing in an arrangement to achieve diagonal air flow (700 µmol s^-1^ in photosynthesis experiment; 1 ml min^-1^ in isoprene measurement). After the leaf is inserted, the chamber can be closed tightly by screws.

**Supplementary Figure S3.** Reference CO_2_ levels detected during measurement of photosynthetic parameters of rust-infected and uninfected black poplar trees at various hours or days post-infection (hpi/ dpi); -1 dpi refers to 1 day before infection.


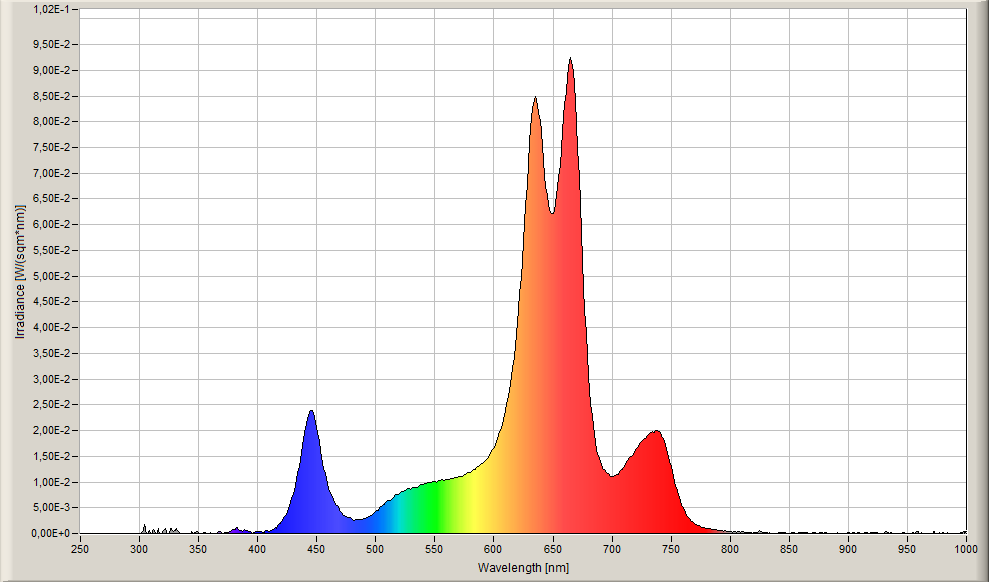


**Supplementary Figure S4.** Light spectrum of the LED lamp used for photosynthesis measurements and isoprene emission analysis. Light intensity on the analyzed leaf was 850 PAR.

**Supplementary Figure S5.** Intercellular CO2 level in leaves of rust-infected black poplar trees (filled symbols) and uninfected controls (open symbols) at different time points after infection (dpi = days post-infection, hpi = hours post-infection; -1 dpi = 1 day before infection). Measurements were made on the second mature leaf counting from the apex. Shown are means ± SEM (*n* = 6). Repeated measures ANOVA yielded no significant effect of either time, rust infection or the interaction of both.


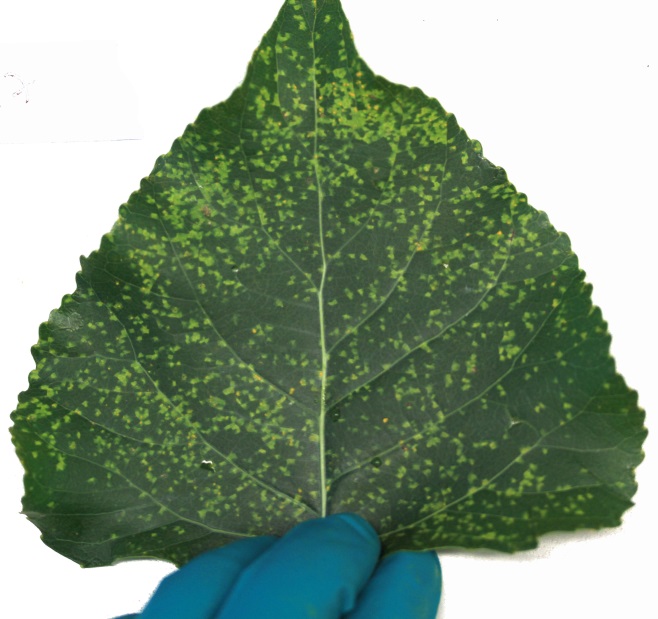


**Supplementary Figure S6**. Leaf of a black poplar tree infected with *Melampsora larici-populina* used for the analysis of isoprene emission at 10 dpi. Chlorotic and yellow lesions are visible around the rust pustules, but no necrosis is seen.

## Supplementary Tables

**Supplementary Table S1.** Carotenoid levels in uredospores of the rust fungus *Melampsora larici-populina* (picture right side). Spores were carefully collected with a brush and scalpel from artificially infected poplar trees. Extraction and analysis was done as described for plant tissue, except for additional grinding (in aluminum tube with steel balls, 5 min x 3, 900 strokes min^-1^) prior to extraction. Presented is the mean ± SEM (*n* = 3 independent spore collections) in mg g^-1^fresh weight. nd – not detected.

|  | β-Carotene | Lutein | Neoxanthin | 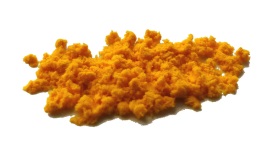Violaxanthin |
| --- | --- | --- | --- | --- |
| Rust fungus spores | 2.17 ± 0.38 | nd | nd | nd |

**Supplementary Table S2.** Genes encoding enzymes of isoprenoid biosynthesis that were found in the transcriptome of rust-infected black poplar leaves, but were annotated as genes of *Melampsora* *larici-populina.* In this rust fungus, like all fungi, the mevalonate pathway is the sole pathway of isoprenoid biosynthesis. The mevalonate pathway enzymes are listed by their Enzyme Commission codes (left panel) and abbreviations (for full names, see Figure 5 legend). Shown is mean ± SEM (*n* = 4) of total raw count of contigs in infected leaves; these contigs were absent in uninfected control leaves.

| Contig | Enzyme Codes | Enzyme Abbreviation | Total raw count |
| --- | --- | --- | --- |
| C62979 | EC 2.3.3.10 | HMGS | 30.0 ± 6.8 |
|  |  |  |  |
| C78714 | EC 1.1.1.3 | HMGR | 13.5 ± 3.1 |
|  |  |  |  |
| C78818 | EC 2.7.1.36 | MK | 1.5 ± 1.2 |
| C79234 | EC 2.7.1.36 | MK | 11.3 ± 5.5 |
| C79468 | EC 2.7.1.36 | MK | 6.5 ± 3.7 |
| C79542 | EC 2.7.1.36 | MK | 3.8 ± 0.9 |
|  |  |  |  |
| C80675 | EC 2.7.4.2 | PMK | 2.0 ± 0.9 |
| C79192 | EC 2.7.4.2 | PMK | 3.3 ± 1.7 |
| C43752 | EC 2.7.4.2 | PMK | 4.0 ± 2.1 |
|  |  |  |  |
| C60670 | EC 4.1.1.33 | PPMD | 12.0 ± 4.4 |
|  |  |  |  |
| C79769 | EC 5.3.3.2 | IDI | 6.8 ± 2.3 |
| C79071 | EC 5.3.3.2 | IDI | 5.8 ± 2.0 |


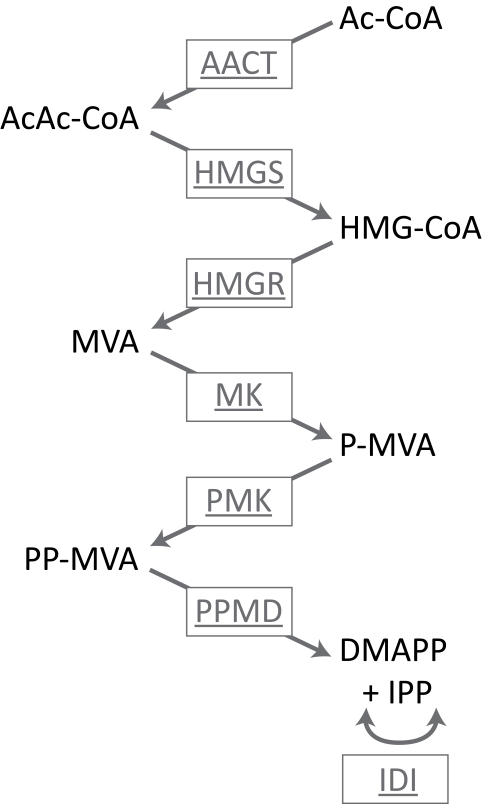

Supplement: Supplementary file 1 [file Table_1.DOCX]
